# Effect of Sonication Treatment and Maceration Time in the Extraction of Polysaccharide Compounds during Red Wine Vinification

**DOI:** 10.3390/molecules26154452

**Published:** 2021-07-23

**Authors:** Leticia Martínez-Lapuente, Zenaida Guadalupe, Paula Pérez-Porras, Ana Belén Bautista-Ortín, Encarna Gómez-Plaza, Belén Ayestarán

**Affiliations:** 1 Instituto de Ciencias de la Vid y del Vino (Universidad de la Rioja, Gobierno de La Rioja y CSIC), Finca La Grajera, 26007 Logroño, Spain; leticia.martinez@unirioja.es (L.M.-L.); zenaida.guadalupe@unirioja.es (Z.G.); belen.ayestaran@unirioja.es (B.A.); 2 Department of Food Science and Technology, Faculty of Veterinary Science, University of Murcia, Campus de Espinardo, 30100 Murcia, Spain; paula.perez2@um.es (P.P.-P.); anabel@um.es (A.B.B.-O.)

**Keywords:** red winemaking, monosaccharides, polysaccharide families, cavitation process, pomace contact times

## Abstract

The application of high-power ultrasounds (US) at 28 kHz to the crushed grapes and the use of different pomace contact times caused changes in the content and composition of monosaccharides and polysaccharides in the musts and wines. These differences were maintained from the moment of pressing (end of maceration) until the end of the alcoholic fermentation. The US increased the content of monosaccharides and polysaccharides in the musts by facilitating their extraction from the solid parts during maceration. The application of medium maceration time (3 days) to sonicated grapes led to an extraction of polysaccharides rich in arabinose and galactose, rhamnogalacturonan type II (RG-II) and mannoproteins (MP), similar to that observed in the control wines made with an extended maceration of 7 days (968.21 vs. 1029.45; 895.04 vs. 1700.50; 356.81 vs. 343.95, respectively). This fact was attributed to a higher extraction in the must during the sonication process and to an important release of pectic polysaccharides during the pressing of the sonicated pomace, which is reported here for the first time. Therefore, the US technology could be useful for increasing the polysaccharide content in the wines or for reducing the maceration time needed to achieve certain levels of wine polysaccharides.

## 1. Introduction

Wine composition depends mainly on the selective extraction of grape components during the winemaking process. Among the macromolecules of enological interest in wines, a lot of attention has been paid to polysaccharides. Wine polysaccharides have their origin in grape skin and pulp cell walls, and cell walls from yeasts. Wine polysaccharides can modulate the astringency and hotness perception in wines, increasing the sweetness sensation and body [1,2], and they are able to interact with wine volatile compounds [3] and thus affect the aroma of the wines.

The extraction of the grape polysaccharides results from the rupture of the grape cell walls that act as a diffusional barrier for the extraction of important metabolites (sugars, acids, volatiles, pigments) and polymers (tannins) contained within the cells into the fermenting must [4].

At the first stages of winemaking, polysaccharides arise from the degradation of the grape cell walls. Several models have been proposed to explain the cell wall architecture [5,6,7], but to date, there seems to be no definitive evidence favoring a given model over the other [8]. Realistic wall models should consider a highly cross-linked wall in which pectin-pectin, pectin-xyloglucan, pectin-cellulose, pectin-phenolics, pectin-protein and xyloglucan-cellulose provide a cohesive network. Type-I cell walls, according to Carpita and Gibeaut [9], are composed of approximately 90% polysaccharides [10] from three major classes that form their structural elements: cellulose, matrix cross-linking glycans (hemicelluloses) and pectic polysaccharides. Cellulose is a linear polysaccharide consisting of long unbranched β-1,4-linked cellobiose chains. Xyloglucans are the predominant hemicelluloses in the dicot primary cell walls. Other hemicelluloses include mannans (β-1,4-mannose backbone, with or without galactose linked by an α-1,6 bond), including glucomannans, galactomannans and galactoglucomannans as well as xylans. Structural classes of pectins include homogalacturonans (HL), rhamnogalacturonans-I (RG-I), rhamnogalacturonans-II (RG-II) and, arabinans, arabinogalactans, arabinans, arabinogalactans and arabinogalactan proteins.

The extraction of all these compounds begins at crushing and requires a maceration time to reach an important concentration in wines [11], and this concentration is also dependent on the skin composition [12], the duration and temperature of maceration [13], the generation of alcohol from alcoholic fermentation [14], and, probably, the cap-management practices and the sulfur dioxide content. The extractability of polysaccharides also increases with the grape maturity [14,15].

The time of maceration is one of the main factors affecting the polysaccharide content in wines. Gil et al. [13,14] showed that the concentration of polysaccharides in Tempranillo and Cabernet Sauvignon wines was higher when the maceration was longer (4 weeks) compared with macerations of 1, 2 and 3 weeks. In Shiraz wines, the concentration of polysaccharides was more influenced by the maceration time than by the application of accentuated cut edges techniques to the grapes [16], while the cryomaceration increased the concentration of polysaccharides in red wines [17].

The presence of several endogenous and, sometimes, exogenous enzymes also helps with the release of polysaccharides from the pectic network of the berry cell walls [11], changing their composition and concentration in the wines.

The use of high-power ultrasounds (US), a non-thermal physical technology that helps to degrade cell walls, could achieve a similar effect to that observed with exogenous enzymes. This technology has been recently approved by the International Organization of Vine and Wine [18]. US technology has shown very positive effects in increasing the content of phenolics and some volatile compounds of sensory relevance in wine, and in reducing the maceration time needed to achieve high phenolic and volatile concentrations in red wines [19,20]. However, little is known on the effect of US on wine polysaccharide content, applied to crushed grapes at a winery-scale. A recent study (Martínez-Lapuente et al. [21]) analyses the polysaccharide content of bottled wines elaborated from sonicated grapes. It concludes that US significantly increases the content of monosaccharides and polysaccharides rich in arabinose and galactose (PRAG) and that wines elaborated with sonicated grapes and 3 days of skin maceration show a polysaccharide and monosaccharide content similar to the control wine elaborated with 7 days of skin maceration. This study confirms that the application of US is an oenological technique able to enhance the breaking down of grape berry cell walls and thus facilitates the incorporation of monosaccharides and polysaccharides into the wines.

The study by Martínez-Lapuente et al. [21] focuses on bottled wines after racking, stabilization processes and bottling. However, the former study did not analyze the effect of different maceration times or the use of ultrasound on the composition and concentration of polysaccharides from the starting must to the end of maceration, where we expected that the composition of both non-sonicated and sonicated musts might be clearly affected by the length of maceration and the level of alcohol at this moment. It neither analyzed the evolution of the extracted polysaccharides from pressing (final of maceration) to the end of alcoholic fermentation, before racking and stabilization processes.

Therefore, the present paper studies, for the first time, the evolution of polysaccharides from crushing to the end of the alcoholic fermentation and how this evolution is affected by the use of US and different maceration times (2 or 3 days).

## 2. Results and Discussion

### 2.1. Changes of Monosaccharide Composition of Musts, Must-Wine at the End of the Maceration and Wine at the End of Alcoholic Fermentation

The evolution of the monosaccharide composition from the crushing of grapes to the end of alcoholic fermentation showed, for all the wines, an increase in the concentration of total pectic monosaccharides (2*-O-*CH_3_-fucose, 2*-O-*CH_3_-xylose, apiose (Api), 2-keto-3-deoxyoctonate ammonium salt (Kdo), galactose (Gal), arabinose (Ara), rhamnose (Rha), galacturonic acid (GalA) and glucuronic acid (GluA)) and of mannose (Man), and a decrease in the concentration of total glucosyl monosaccharides derived from cellulose and hemicellulose (xylose (Xyl), fucose (Fuc) and glucose (Glc)). The maceration time, sonication and alcoholic fermentation was the main process affecting this content (Table 1 and Table 2). The treatments applied in this study (sonication of the crushed grape and different maceration times) caused important changes in the glycosyl residues between the control and sonicated samples during the period between the initial must and the moment of pressing and between the pressing and the end of alcoholic fermentation.

The work of Martínez-Lapuente et al. [21] described a significant increment in the total pectic monosaccharides in sonicated musts, which proved the disruption of the grape berry cell wall polysaccharides caused by the sonomechanical effect of ultrasounds. Ultrasound treatment led to a weakening in the crosslinking wall polymers such as pectin-pectin, pectin-xyloglucan, pectin-cellulose, pectin-phenolics and xyloglucan-cellulose [8], increasing the presence of the disrupted cell wall polymers in the must and facilitating the extraction of grape cell compounds, as anthocyanins and tannins, which increased color and total phenol content in the sonicated must [19,21]. Pérez-Porras et al. [19], using optical microscopy techniques, confirmed the direct effect of US on the degradation and morphological modification of the external layers of skin.

A reduction of more than 75% in the total content of glucosyl monosaccharides (glucose, xylose and fucose) was observed in all the elaborations at the end of the maceration period; in the case of S28MF-2d and S28MF-3d, the reduction was significant compared with S28-M (Table 1 and Table 2). This fact was due to the reduction of glucose (Glc), suggesting that the solubilization of structural polysaccharides from grape cell walls was limited due to the enzymatic activity and/or ethanol content formed during the alcoholic fermentation. As the maceration—time increased, which meant a longer time for the pomace to be in contact with an increasing concentration of ethanol, the abundance of Glc decreased (Table 1).

Therefore, at the moment of pressing, the glucose content in short maceration must-wines was the highest, followed by samples with a medium maceration and, at a distance, those with a 7-day maceration (the average density values were 1.109; 1.082 and 1.008 g/L, respectively, for short, medium and long maceration must-wines). It is interesting to note that, in sonicated must-wines, with a density similar to their controls (the 20/20 density value of S28MF-2d and CMF-2d was 1.108 and 1.110 g/L, respectively; the 20/20 density value of S28MF-3d and CMF-3d was 1.079 and 1.084 g/L, respectively) and thus with similar ethanol content, the glucose (Glc) and xylose (Xyl) values were significantly higher than their respective controls. These results were probably due to the direct effect of US treatment on the disruption of the xyloglucan-cellulose, pectin-xyloglucan and pectin-cellulose network.

A reduction of more than 75% in the total content of glucosyl monosaccharides (The content of the major monosaccharide components derived from the pectic polysaccharides in the must-wines) was higher at the end of the maceration period with respect to the initial must, and this increase was more evident as the maceration time increased (Table 1 and Table 2). This was an expected result, since longer maceration-fermentation times favored the extraction yield of soluble polysaccharides from the flesh and skin tissues, helped by natural enzymatic cocktails that degrade cell walls, a natural process that occurs during maceration and was previously reported by other authors [22].

At the moment of pressing, the content of the major monosaccharides and pectic components (arabinose (Ara), galactose (Gal), galacturonic acid (GalA) and rhamnose (Rha)) was higher than that of non-pectic components, such as Xyl and Fuc. This result was probably due to the higher stabilization of the pectic polysaccharides in the hydroalcoholic medium formed during the alcoholic fermentation [23]. The origin of mannose (Man) is unclear [24]. A previous study demonstrated that mannoprotein concentration in wines increased in the last stages of fermentation [11]. In this sense, it was difficult to elucidate the origin of Man in must-wines with short or medium maceration time, whereas in CMF-7d, with a 20/20 density of 1.000 g/L, part of the Man content probably came from the mannoproteins of the yeast.

The total content of pectic monosaccharides (Gal, Ara, GalA, GluA, Rha, 2*-O-*CH_3_-fucose, 2*-O-*CH_3_-xylose, Api and Kdo) of the sonicated must-wines was significantly higher than their controls at the end of the maceration time (Table 1). The difference in the total content of pectic monosaccharides between S28MF-3d and S28MF-2d with respect to CMF-7d was 34% and 56%, respectively, and these differences were greater in CMF-3d (49%) and in CMF-2d (71%).

These results indicated that the sonication of the grapes followed by a three-day maceration was a very effective treatment for the extraction of pectic polysaccharides from grapes, and the results achieved were the closest to that obtained in CMF-7d. Galactose (Gal) was the major pectic monosaccharide in all the must wines, followed by arabinose (Ara) and galacturonic acid (GalA).

Important changes were observed in the extraction and solubilization of monosaccharides from the moment of pressing (end of maceration) to the end of alcoholic fermentation. The total content of glucosyl monosaccharides continued significantly decreasing (more than 80%, except in CW7d, which was 45%), due to a significant reduction in glucose (Glc) (Table 1 and Table 2). Guadalupe and Ayestarán [11] showed that an important amount of grape structural glucosyl polysaccharides were extracted during alcoholic fermentation, although their solubilization was limited, and more than 60% of these compounds were unstable and precipitated.

However, one important finding that has not been previously reported was the significant increase in the total content of pectic monosaccharides in sonicated must-wines from the moment of pressing to the end of alcoholic fermentation, probably due to the release of pectic polysaccharides from the flesh and skin tissues from the pomace at the pressing stage [25], an effect not observed during the pressing of control must-wines. These results confirmed that the pomace pressing was more effective in releasing the grape berry cell wall polysaccharides from sonicated-macerated grapes than from non-sonicated grapes.

It is important to highlight that the majority RG-II markers (2*-O-*CH_3_-fucose, 2*-O-*CH_3_-xylose, 2-keto-3-deoxyoctonate ammonium salt and apiose) [26,27,28] of short and medium maceration wines significantly increased from the moment of pressing, and they were higher in sonicated wines than in their controls (Table 1 and Table 2). The highest increase was observed in medium maceration wines. The RG-II content in skin tissue is three-fold higher than that on pulp tissue [29]. The pressing of the pomace probably facilitated the release of RG-II from the cell walls of the skin and from the flesh attached to the skin.

An increase in the mannose (Man) content was also observed in the wines at the end of the maceration, reaching similar concentrations in CW-7d and S28W-3d (Table 1 and Table 2). In the later stages of fermentation, most of the Man content probably comes from the mannoproteins of the yeast.

Important differences were observed at the end of alcoholic fermentation in the content of total monosaccharides (Table 1). The long-macerated wine (CW-7d) stood out for its significant high value in the total content of pectic monosaccharides. However, the content of galactose (Gal), the major monosaccharide of Monastrell wines [17,30], was similar in CW-7d and in sonicated wines (S28MF-3d and S28MF-2d). Arabinose (Ara) and galacturonic acid (GalA) were the following major monosaccharides in the wines. The Ara and Rha content in CW-7d were significantly higher than in the other wines, while the GalA content did not present significant differences between the wines, except for CW-2d.

In sonicated wines, the galactose (Gal) and arabinose (Ara) contents were significantly higher than in their controls, while galacturonic acid (GalA) and glucuronic acid (GluA) only showed significant differences in S28W-2d. Therefore, the difference in the total content of pectic monosaccharides between SW28-3d and SW28-2d with respect to CW-7d was only 15% and 23%, respectively, while the difference between the control wines of medium and short maceration compared with CW-7d was higher (35% and 49%, respectively). These results indicated that US cavitation caused the disruption of the cell walls of crushed grapes and facilitated the release to the hydroalcoholic medium of the flesh tissue from the pomace at the pressing stage. Therefore, the content of pectic monosaccharides in US medium macerated red wines was similar to that of long macerated wines.

### 2.2. Changes in the Polysaccharide Families of Musts, Must-Wine at the End of the Maceration and Wines at the End of Alcoholic Fermentation

The main polysaccharides present in musts, must-wines at the moment of pressing and finished wines were grouped in four major families: (i) polysaccharides rich in arabinose and galactose (PRAG) (arabinogalactans type I, AG-I, and arabinogalactans type II joined to protein, AGP); (ii) rhamnogalacturonans (rhamnogalacturonans type I, RG-I, and rhamnogalacturonans type II, RG-II); (iii) homogalacturonans (HL), all of which arise from the pectocellulosic portion of the cell walls of grape berries; and (iv) mannoproteins (MP)/mannans, arising from mannans in the grape and yeast, as well as mannoproteins present in the yeast cell walls [31].

The concentration of the different polysaccharide families was monitored during the maceration-fermentation process (Table 3 and Table 4), and the results obtained showed good agreement with the observations described in the previous section.

The rate of extraction and solubilization of polysaccharide families differed depending on the polysaccharide family. Polysaccharides rich in arabinose and galactose (PRAG), which include soluble arabinogalactans type II joined to protein (AGP) [29], were easily extracted by crushing, followed by rhamnogalacturonans type II (RG-II) and homogalacturonans (HL) and, at a distance, by mannoproteins (MP)/mannans. The US treatment significantly increased the extraction of most of the polysaccharide families from the grape cell walls (Table 3), as previously reported by Martínez-Lapuente et al. [21].

During the pomace maceration period, a significant increase in the content of PRAG, RG-II, MP/Mannans and total polysaccharide families (PST) was observed, although the increase was no significant for RG-II in S28MF-3d (Table 3 and Table 4). This increase was attributed to the disruption of the skin and pulp cell walls, which also facilitated the extractability of anthocyanidins and tannins into the must-wine [32,33].

The concentration of mannoproteins (MP)/mannans did not show significant differences between CMF-7d and S28MF-3d, which showed the highest values. The 20/20 density value of S28MF-3d indicated that the yeasts were in an exponential phase of growth, a metabolic phase that probably influenced the release of MP of yeast [11,34]. This fact, together with the cavitation-US effect in the mannans of the grape cell walls, could explain the high content of MP/mannans in the S28MF-3d sample.

The rhamnogalacturonans type II (RG-II) value was significantly higher in CMF-7d than in wines with shorter maceration times. RG-II is more tightly bound to the cell wall matrix of the grape cell walls [11] and probably needs more maceration time to be extracted and solubilized.

At the end of the maceration, the content of polysaccharides rich in arabinose and galactose (PRAG) of S28MF-3d and S28MF-2d was significantly higher than their respective controls (CMF-3d and CMF-2d) (Table 3). It is interesting to note that US technique was highly effective for disrupting cell walls. In addition to the release of polysaccharide compounds, it increased the extraction of anthocyanins and tannins (Pérez Porras et al. [19]), confirming that the breakdown of the cell wall structures produced by US had a positive effect on the extraction of phenolic components [19,35,36,37].

From the end of the maceration (2 or 3 days) to the end of alcoholic fermentation, the content of PRAG, RG-II, MP/Mannans, HL and total polysaccharide families (PST) significantly increased in sonicated samples (Table 3 and Table 4). On the contrary, the increase in PRAG for CW-7d, CW-3d and CW-2d and in RG-II and PST for CW-7d was not significant. The pressing process of the sonicated pomace was very effective in releasing the pulp adhered to the pomace. The suspension of a greater amount of residual pulp and skin tissue in the must-wine favored the increase in all polysaccharide families, from the most easily extractable (PRAG) to the one most attached to the matrix of the cell wall of the grape cell walls (RG-II). Consequently, at the end of alcoholic fermentation, the wines from sonicated grapes showed significantly higher content of PRAG, RG-II and PST than their respective controls.

The composition profile of the polysaccharide families was similar in the wines (Table 3). PRAG and RG-II was the major family followed by MP and, distantly, HL. It is important to highlight that the content of PRAG was similar in control wines from long maceration time (7 days) and sonicated wines from short and medium maceration time (2 and 3 days). The RG-II content in wines from long maceration times was closer to that of wines from sonicated grapes and medium maceration time. CW-7d, S28W-3d and CW-3d wines did not show significant differences in the MP content (Table 3) since most of the MP arises from yeasts, which were not affected by sonication.

The results suggested that the content of the polysaccharide families in wine depends on the extraction and solubility of the concrete family, but also depends on the treatments that degrade the cell wall structure of the grape, such as cavitation-US, maceration time and pressing of the sonicated pomace. Considering the concentration of PRAGs, RG-II and MP reported in bottled wines by Martínez-Lapuente et al. [21], a decrease in PRAGs and RGII is observed due to the racking and stabilization processes, with the MP being less affected than the rest of wine polysaccharides. However, the same differences described in the present paper due to sonication and maceration time were maintained in bottled wines.

### 2.3. Principal Factors of Variability of the Content of Wine Monosaccharides and Polysaccharide Families

A multivariate analysis of variance (MANOVA) was conducted in the wine samples to analyze the effect of maceration time (short and mid) and sonication on the wine monosaccharides and polysaccharide families (Table 5).

The factor maceration time and maceration time × sonication accounted for a small fraction of the observed variation, whereas the sonication effect was the dominant factor of variation for most of the monosaccharide and polysaccharide concentration (Table 5). Except for Rha, Fuc and Xyl, sonication had a great effect on the average concentration of monosaccharides and polysaccharides, confirming the higher extraction in US samples.

Regarding maceration time, the wines obtained after 3 days of skin maceration time presented greater content of 2*-O-*CH_3_-Fuc, 2*-O-*CH_3_-Xyl, Ara, Rha, GalA, Fuc, Man, RG-II, MP and PST than those with a short skin maceration time. However, the apiose and homogalacturonan content decreased with the maceration time. When the grapes were sonicated at 28 kHz, the resulting wines presented higher content of most of pectic monosaccharides (except for Rha and Gal), total pectic monosaccharides, Glc, total glucosyl monosaccharides, PRAG, RG-II, HL, MP and total polysaccharide families. The effect of the maceration time × sonication interaction was significant for 2-*O*-CH_3_-Fuc, 2*-O-*CH_3_-Xyl, Api, Ara, Glc, total glucosyl monosaccharides, RG-II and HL, and it was the dominant factor in the variation of HL content.

## 3. Materials and Methods

### 3.1. Chemicals

All reagents were analytical grade unless otherwise stated. Standards of different monosaccharides were used to perform the calibration curves. D-(+)-Fucose > 98%, L-rhamnose monohydrate > 99%, 2*-O-*methyl D-xylose > 99%, L-(+)-arabinose > 99%, D-(+)-xylose > 99%, D-(+)-galactose > 99%, D-(+)-glucose 99.5%, D-(+)-mannose ≥ 99% and Kdo (2-keto-3-deoxy-D-manno-octulosonic acid) ≥ 97% were supplied by Sigma (Beerse, Belgium); and D-(+)-galacturonic acid > 93%, D-glucuronic acid ≥ 97% and myo-Inositol ≥ 98% (internal standard) were obtained from Fluka (Buch, Switzerland).

Ethanol 96% (*v/v*), hexane-(n) 99+%, HPLC grade and acetyl chloride ≥ 98.0% were supplied by Merck (Darmstadt, Germany); and methanol anhydrous 99.8%, pyridine 99.5+%, hexamethyldisilazane ≥ 99.0% and trimethylchlorosilane ≥ 98.0% were supplied by Merck (Darmstadt, Germany).

### 3.2. Equipments

Samples were centrifuged using a Sorvall Lynx 4000 refrigerated centrifuge (Thermo Scientific, Barcelona, Spain). pH measurements were performed with a sensION 3 pH meter (Hach Lange GmbH Headquarter, Düsseldorf, Germany). Gas chromatography–mass spectrometry (GC–MS) was performed using an Agilent Technologies 7890A (Agilent Technologies, Waldbronn, Germany) chromatograph with a Chemstation Agilent software data-processing software, equipped with a 7653B automatic injector coupled to a 5975C VL quadrupole mass detector (MS). The different monosaccharides were quantified in selected ion monitoring (SIM) mode, selecting the appropriate number of ions for each compound (*m/z*). D-galacturonic acid, L-rhamnose, L-fucose, D-galactose, D-glucose, D-mannose and D-xylose with 204 ion; and D-glucuronic acid, L-arabinose, Kdo, 2*-O-*methyl-L-fucose, Dha and aceric acid with 217 ion; 2*-O-*methyl D-xylose with 146 ion; apiose with 191 ion; and myo-inositol (internal standard) with 305 ion.

### 3.3. Vinification and Sample Collection

Approximately 1400 kg of red grapes from Monastrell variety were harvested on the vintage 2019 from a plot in Jumilla (Murcia, Spain) once the optimum technological maturity was reached (14 ºBaumé). Grapes were destemmed and crushed and divided into two batches. One batch was left untreated as control and another batch was subjected to ultrasound treatment using a frequency of 28 kHz in a pilot-scale high-power ultrasound equipment (MiniPerseo, Agrovin S.A., Alcazar de San Juan, Spain). The system operated at 2500 W with a power density of 8 W/cm^2^, treating 400 kg of crushed grapes per hour.

Five types of elaboration were carried out: three control vinifications using untreated crushed grape with 2 (CMF-2d), 3 (CMF-3d) and 7 (CMF-7d) days of maceration and two vinifications using ultrasound-treated crushed grape with 2 (S28MF-2d) and 3 days of maceration (S28MF-3d). Triplicates of each elaboration were made, using a total of fifteen 50 L stainless steel tanks. The crushed grape was distributed into the tanks maintaining the same pomace solid/liquid ratio. Total acidity correction was made up to 5.5 g/L of tartaric acid, and a commercial *Saccharomyces cerevisiae* yeast was added at a dose of 0.20 g/kg (Viniferm CT007, Agrovin, Alcázar de San Juan, Spain). Tanks were kept at a controlled room temperature during fermentation (23 ± 2 °C). From the beginning to the end of maceration, the cap was punched down twice a day. After maceration time, the pomaces were pressed in a 75 L pneumatic press. Free-run and pressed must-wines were mixed and stored until completion of the alcoholic fermentation. The fermented wines were named as CW-2d, CW-3d, CW-7d, S28W-2d and S28W-3d. Analysis of must (beginning of maceration, C-M and S-28M), must-wine (end of maceration) and wine (end of alcoholic fermentation) were performed. The basic physico-chemical parameters can be found in [19] and were represented in Table S1 (see Supplementary Material).

### 3.4. Precipitation of Total Soluble Wine Polysaccharides

Polysaccharides of must, must-wine and wine were recovered by precipitation after ethanolic dehydration, as previously described [38,39]. The polysaccharide extraction was performed in triplicate in each sample.

### 3.5. Identification and Quantification of Monosaccharides by GC–MS

The monosaccharide composition of must, must-wine and wine was determined by GC–MS of their trimethylsilyl-ester *O*-methyl glycosyl residues obtained after acidic methanolysis and derivatization, as previously described [38]. The content of each polysaccharide family was estimated from the concentration of individual glycosyl residues, which are characteristic of structurally identified must and wine polysaccharides [39,40]. The content of total polysaccharides families (PST) was estimated from the sum of PRAG, MP, RG-II and HL.

The chromatographic column was a Teknokroma fused silica capillary column (30 m × 0.25 mm × 0.25 mm) of phase 5% phenyl/95% methyl polysiloxane. The oven program started at an initial temperature of 120 °C, which was increased at a rate of 1 °C/min to 145 °C and then to 180 °C at a rate of 0.9 °C/min and, finally, to 230 °C at 40 °C/min. The GC injectors were equipped with a 3.4 mm I.D. liner and were maintained at 250 °C with a 1:20 split ratio. The carrier gas was helium (99.996%) at a flow rate of 1 mL/min. Ionization was performed by electron impact (EI) mode at 70 eV. The temperatures used were 150 °C for the MS Quad, 230 °C for the MS Source and 250 °C for the transfer line.

### 3.6. Statistical Analyses

All the data were expressed as the average of three replicates. One-factor analysis of variance and multivariate analysis of variance were performed using the SPSS v. 15.0 for Windows statistical package (SPSS Statistics, Inc., Chicago, IL, USA) with post hoc Duncan (*p* < 0.05) to determine the significant differences. Musts, must-wines and wines were compared separately, and must-wines and wines with 2- and 3-day maceration time were also compared separately. Analysis of variance paired-samples *t*-test was made to determine differences between the consecutive stages of winemaking.

## 4. Conclusions

The sonication treatment of the crushed grapes and the different maceration time caused important changes in the content of monosaccharides and polysaccharide families from the starting must to the end of pomace maceration time, and from this stage to the end of alcoholic fermentation. PRAG, RG-II, HL and MP or mannans increased in all samples during the pomace maceration, and the sonication of the grapes especially intensified the extraction and solubility of PRAG during the maceration.

From pressing to the end of alcoholic fermentation, the evolution of the content of the polysaccharide families and PST was different in the sonicated wines from that in the controls. In sonicated wines, the concentration of all families increased, while their concentration did not vary for CW-7d. This effect was attributed to sonication, which facilitated the release of pectic polysaccharides from the flesh tissues from the pomace during the pressing process, a result that was reported for the first time in this study.

The wines made with sonicated grapes presented higher concentration of polysaccharides than their control wines at the end of alcoholic fermentation. Maceration time of 3 days led to wines with a similar polysaccharide profile and content to those achieved in control wines with 7 days of pomace maceration. Therefore, the US technology could be useful for increasing the polysaccharide content in the wines or for reducing the maceration time needed to achieve certain levels of wine polysaccharides.

## Figures and Tables

**Table 1:** Monosaccharide composition (mg/L) of polysaccharides during winemaking ^a^.

Parameter ^b^	Musts ^c^		Maceration-Fermentation ^c^					End of Alcoholic Fermentation ^c^				
	C-M	S28-M	CMF-2d	S28MF-2d	CMF-3d	S28MF-3d	CMF-7d	CW-2d	S28W-2d	CW-3d	S28W-3d	CW-7d
2-OMeFuc	0.89 a	1.14 b	1.35 ab A	0.93 a A	2.19 b α	1.78 ab α	16.20 c	5.06 a A	6.43 a B	6.54 a α	11.22 b β	21.76 c
2-OMeXyl	0.61 a	0.79 b	0.59 a B	0.33 a A	1.08 a α	0.81 a α	7.63 b	2.50 a A	3.61 a B	2.98 a α	5.54 ab β	9.63 b
Api	0.64 a	0.10 a	0.30 a A	0.28 a A	0.54 a α	0.53 a α	2.78 b	1.23 b A	2.40 c B	0.55 a α	0.94 b β	5.51 d
Kdo	nd	nd	0.28 a A	1.09 ab B	0.56 a α	0.64 a α	2.34 b	1.06 a A	1.92 a B	0.85 a α	1.58 a α	4.05 b
Gal	91.12 a	159.48 b	221.17 a A	362.10 b B	458.80 c α	575.30 d β	782.09 e	378.18 a A	632.11 bc B	513.71 ab α	671.13 c β	705.62 c
Ara	28.04 a	59.65 b	58.47 a A	73.93 a B	77.84 a α	141.07 b β	254.32 c	87.79 a A	101.93 a B	88.31 a α	131.39 b β	195.48 c
Rha	9.48 a	17.69 b	24.39 a A	29.36 a A	36.00 a α	43.91 a α	118.71 b	24.68 a A	28.77 ab A	35.52 b α	36.23 b α	95.43 c
GalA	38.51 a	60.84 b	51.49 a A	84.10 b B	65.08 a α	71.18 a b	74.28 ab	62.21 a A	82.44 b B	82.18 b α	90.93 b α	83.01 b
GluA	4.61 a	8.16 b	7.79 a A	11.29 b B	12.89 b α	13.88 b α	23.66 c	14.38 a A	19.50 a B	14.26 a α	15.27 a α	19.03 a
ΣPectic monosaccharides (1)	173.91 a	307.86 b	365.82 a A	563.41 b B	654.98 b α	849.10 c β	1282.00 d	577.07 a A	879.11 bc B	744.90 b α	964.24 c β	1139.52 d
Fuc	0.82 a	1.44 b	1.30 a B	1.00 a A	1.46 a α	1.00 a α	4.93 b	1.96 a A	1.86 a A	2.18 a α	2.72 a α	5.29 b
Xyl	3.88 a	7.16 b	8.28 a A	13.04 b B	10.92 ab α	16.64 c β	20.53 d	11.19 a A	11.44 a A	12.60 a α	12.44 a α	34.93 b
Glc	3289.61 a	3715.03 a	635.26 d A	836.54 e B	235.06 b α	405.38 c β	110.00 a	28.24 a A	70.44 c B	33.84 ab α	50.69 b β	34.05 ab
^e^ΣGlucosyl monosaccharides (2)	3294.32 a	3723.64 a	644.84 d A	850.57 e B	247.44 b α	423.02 c β	135.46 a	41.39 a A	83.74 c B	48.62 ab α	65.85 bc β	74.27 c
Man	9.09 a	17.06 b	42.99 a A	51.90 a A	168.95 b α	245.88 c β	265.89 c	187.76 a A	232.93 b B	266.54 bc α	285.45 c α	275.16 c
Σ1 + Σ2 + Mn	3477.32 a	4048.55 a	1053.65 a A	1465.88 b B	1071.36 a α	1518.00 b β	1683.84 c	806.22 a A	1195.77 bc B	1060.05 b α	1315.53 cd β	1488.95 d
Σ1 + Σ2 + Mn − Glc	187.71 a	333.52 b	418.39 a A	629.34 b B	836.31 c α	1112.62 d β	1573.84 e	777.99 a A	1125.33 bc B	1026.22 b α	1264.85 cd β	1454.90 d

^a^ Average of the three measurements. Different letters indicate statistical differences (*p <* 0.05). Lower-case letters compare separately the winemaking stages. Upper-case letters compare samples with 2-day maceration time. Greek alphabet letters compare samples with 3-day maceration time. ^b^ See abbreviations. ^c^ C-M, control must; S28-M, must with sonicated grapes at 28 kHz; CMF-2d, control must-wine with 2-day maceration; S28MF-2d, 28 kHz-treated must-wine with 2-day maceration; CMF-3d, control must-wine with 3-day maceration; S28MF-3d, 28 kHz-treated must-wine with 3-day maceration; CMF-7d, control must-wine with 7-day maceration; CW-2d, control wine with 2-day maceration; S28W-2d, 28 kHz-treated wine with 2-day maceration; CW-3d, control wine with 3-day maceration; S28W-3d, 28 kHz-treated wine with 3-day maceration; CW-7d, control wine with 7-day maceration. nd: not detected.

**Table 2:** Statistical differences (*p <* 0.05) ^a^ of monosaccharide composition (mg/L) of (**A**) during the winemaking ^b,c^ evolution.

Parameter ^d.^	C-2d ^b^		C-3d ^b^		S28-2d ^b^		S28-3d ^b^		C-7d ^b^	
	M-F ^c^	MF-W ^c^	M-MF ^c^	MF-W ^c^	M-MF ^c^	MF-W ^c^	M-MF ^c^	MF-W ^c^	M-MF ^c^	MF-W ^c^
2-OMeFuc	0.059	0.007	0.067	0.023	0.436	0.001	0.083	0.011	0.002	0.122
2-OMeXyl	0.792	0.013	0.044	0.030	0.049	0.001	0.848	0.017	0.012	0.650
Api	0.249	0.030	0.673	0.777	0.131	0.001	0.003	0.023	0.149	0.129
Kdo	0.059	0.003	0.050	0.242	0.010	0.053	0.018	0.134	0.165	0.253
Gal	0.011	0.182	0.002	0.412	0.021	0.023	0.001	0.056	0.001	0.345
Ara	0.016	0.003	0.001	0.148	0.001	0.036	0.009	0.265	0.012	0.288
Rha	0.011	0.922	0.001	0.935	0.024	0.916	0.011	0.250	0.017	0.260
GalA	0.280	0.173	0.033	0.097	0.043	0.846	0.173	0.022	0.040	0.068
GluA	0.024	0.049	0.018	0.630	0.032	0.042	0.012	0.399	0.003	0.551
ΣPectic monosaccharides (1)	0.019	0.129	0.001	0.251	0.014	0.035	0.002	0.020	0.002	0.323
Xyl	0.062	0.013	0.051	0.506	0.031	0.424	0.012	0.137	0.002	0.008
Fuc	0.006	0.105	0.028	0.017	0.002	0.000	0.250	0.001	0.006	0.752
Glc	0.089	0.002	0.071	0.011	0.005	0.001	0.003	0.001	0.066	0.024
ΣGlucosyl monosaccharides (2)	0.089	0.002	0.071	0.011	0.005	0.001	0.003	0.002	0.067	0.038
Man	0.023	0.003	0.011	0.015	0.013	0.003	0.003	0.160	0.004	0.818
Σ1 + Σ2 + Mn	0.100	0.153	0.102	0.820	0.004	0.030	0.005	0.019	0.169	0.341
Σ1 + Σ2 + Mn − Glc	0.009	0.054	0.002	0.092	0.014	0.017	0.000	0.024	0.002	0.499

^a^*p* value obtained by analysis of variance paired-samples *t*-test of the same winemaking. ^b^ C-2d, control vinification with 2-day maceration; C-3d, control vinification with 3-day maceration; S28-2d, sonicated vinification at 28 kHz with 2-day maceration; S28-3d, sonicated vinification at 28 kHz with 3-day maceration; C-7d, control vinification with 7-day maceration. ^c^ M, must; MF, final of maceration-fermentation; W, wine at the end of the alcoholic fermentation. ^d^ See abbreviations.

**Table 3:** Concentration of polysaccharide families (mg/L) during winemaking ^a^.

Parameter ^b^	Musts ^c^		Maceration-Fermentation ^c^					End of Alcoholic Fermentation ^c^				
	C-M	S28-M	CMF-2d	S28MF-2d	CMF-3d	S28MF-3d	CMF-7d	CW-2d	S28W-2d	CW-3d	S28W-3d	CW-7d
RG-II	76.80 a	98.24 b	105.10 ab B	70.13 a A	174.73 b α	139.97 ab α	1280.85 c	403.59 a A	527.02 a B	514.15 a α	895.04 b β	1700.50 b
PRAG	144.22 a	265.33 b	346.17 a A	551.33 b B	677.15 c α	897.59 d β	1215.15 e	565.60 a A	909.21 bc B	739.83 ab α	968.21 bc β	1029.45 c
HL	30.47 a	50.58 a	39.37 a A	75.71 c B	45.37 a α	55.15 b β		16.69 a A	24.55 b B	23.29 b		
MP/mannans	11.36 a	21.33 b	53.74 a A	64.87 a A	211.19 b α	307.35 c β	332.36 c	234.70 a A	291.16 b B	333.17 bc α	356.81 c α	343.95 c
PST	262.85 a	435.48 b	544.37 a A	762.04 a A	1108.44 b α	1400.05 c β	2828.36 d	1220.59 a A	1751.94 ab B	1610.45 a α	2220.06 b β	3073.90 c

^a^ Average of the three measurements. Different letters indicate statistical differences (*p* < 0.05). Lower-case letters compare separately the musts, the must-wines at the end of maceration and the wine samples. Upper-case letters compare samples with 2-day maceration time. Greek alphabet letters compare samples with 3-day maceration time. ^b^ See abbreviations. ^c^ C-M, control must; S28-M, must with sonicated grapes at 28 kHz; CMF-2d, control must-wine with 2-day maceration; S28MF-2d, 28 kHz-treated must-wine with 2-day maceration; CMF-3d, control must-wine with 3-day maceration; S28MF-3d, 28 kHz-treated must-wine with 3-day maceration; CMF-7d, control must-wine with 7-day maceration; CW-2d, control wine with 2-day maceration; S28W-2d, 28 kHz-treated wine with 2-day maceration; CW-3d, control wine with 3-day maceration; S28W-3d, 28 kHz-treated wine with 3-day maceration; CW-7d, control wine with 7-day maceration.

**Table 4:** Statistical differences (*p* < 0.05) ^a^ of polysaccharide families (mg/L) of (**A**) during the winemaking ^b,c^ evolution.

Parameter ^d^	C-2d ^b^		C-3d ^b^		S28-2d ^b^		S28-3d ^b^		C-7d ^b^	
	M− MF ^c^	MF-W ^c^	M− MF ^c^	MF-W ^c^	M− MF ^c^	MF-W ^c^	M− MF ^c^	MF-W ^c^	M− MF ^c^	MF-W ^c^
RG-II	0.000	0.001	0.009	0.002	0.049	0.000	0.076	0.002	0.002	0.083
PRAG	0.000	0.135	0.000	0.191	0.019	0.020	0.001	0.002	0.001	0.129
HL	0.294	0.001	0.146	0.002	0.001	0.007	0.323	0.000	0.074	
MP/mannans	0.018	0.001	0.011	0.012	0.013	0.000	0.002	0.014	0.003	0.340
PST	0.000	0.023	0.001	0.004	0.024	0.004	0.002	0.001	0.001	0.367

^a^*p* value obtained by analysis of variance paired-samples *t*-test of the same winemaking. ^b^ C-2d, control vinification with 2-day maceration; C-3d, control vinification with 3-day maceration; S28-2d, sonicated vinification at 28 kHz with 2-day maceration; S28-3d, sonicated vinification at 28 kHz with 3-day maceration; C-7d, control vinification with 7-day maceration. ^c^ M, must; MF, final of maceration-fermentation; W, wine at the end of the alcoholic fermentation. ^d^ See abbreviations.

**Table 5:** MANOVA statistical analysis and percentage of attributable variance (%) of the independent effect of maceration time (MF) and sonication (S) and the interaction of both of them (MF × S) in wine samples.

Parameter ^a^	Maceration Fermentation Time (MF)				Sonication (S)				Interactions		
	2 Days	3 Days	*p-Value*	MF (%)	C	S28	*p*	S (%)	*p*	MF × S (%)	Residual (%)
2-OMeFuc	5.74	8.88	0.000	41.98	5.80	8.82	0.000	39.07	0.007	11.62	7.34
2-OMeXyl	3.05	4.26	0.003	24.19	2.74	4.57	0.000	55.58	0.040	8.70	11.53
Api	1.82	0.74	0.000	58.33	0.89	1.67	0.000	31.29	0.001	7.85	2.53
Kdo	1.49	1.22	0.178	7.55	0.95	1.75	0.003	64.27	0.721	0.47	27.71
Gal	505.14	592.42	0.091	11.08	445.95	651.62	0.002	61.55	0.319	3.39	23.98
Ara	94.86	109.85	0.012	15.80	88.05	116.66	0.000	57.56	0.014	14.72	11.91
Rha	26.73	35.88	0.026	45.70	30.10	32.50	0.498	3.13	0.631	1.55	49.62
GalA	72.32	86.55	0.002	38.57	72.19	86.68	0.002	40.00	0.106	6.27	15.16
GlcA	16.94	14.76	0.122	15.24	14.32	17.39	0.040	30.43	0.141	13.60	40.73
ΣPectic monosaccharides (1)	728.09	854.57	0.031	15.34	660.99	921.67	0.001	65.17	0.416	1.64	17.85
Fuc	1.91	2.45	0.023	39.32	2.07	2.29	0.279	6.66	0.126	14.44	39.58
Xyl	11.31	12.52	0.369	10.13	11.90	11.94	0.974	0.01	0.873	0.30	89.55
Glc	49.34	42.26	0.065	4.28	31.04	60.56	0.000	74.51	0.005	13.73	7.48
ΣGlucosyl monosaccharides (2)	62.56	57.23	0.220	2.36	45.00	74.79	0.000	73.83	0.014	13.12	10.68
Man	210.34	275.99	0.000	72.49	227.15	259.19	0.002	17.26	0.114	2.90	7.35
Σ1 + Σ2 + Mn	1001.00	1187.79	0.010	20.80	933.14	1255.65	0.000	62.01	0.259	2.68	14.51
Σ1 + Σ2 + Mn− Glc	951.66	1145.53	0.007	25.21	902.10	1195.09	0.001	57.59	0.337	1.98	15.21
RG-II	465.30	704.60	0.000	39.67	458.87	711.03	0.000	44.05	0.002	11.48	4.80
PRAG	737.41	854.02	0.092	10.59	652.72	938.71	0.002	63.67	0.372	2.58	23.16
HL	20.62	11.65	0.000	20.41	20.00	12.28	0.000	15.09	0.000	61.42	3.07
MP	262.93	344.99	0.000	72.49	283.94	323.98	0.002	17.26	0.114	2.90	7.35
PST	1486.26	1915.25	0.002	31.68	1415.52	1986.00	0.000	56.02	0.687	0.26	12.04

^a^ See abbreviations.

## Data Availability

Not applicable.

## Supplementary Materials

The following are available online, Table S1. Standard enological parameters of must, must-wines at the end of maceration and wine samplesª.

## Abbreviations

2-OMeFuc	2*-O-*CH_3_-fucose
2-OMeXyl	2*-O-*CH_3_-xylose
Api	Apiose
AGP	Arabinogalactans type II joined to protein
Ara	Arabinose
Fuc	Fucose
Gal	Galactose
GalA	Galacturonic Acid
Glc	Glucose
GluA	Glucuronic Acid
HL	Homogalacturonans
Kdo	2-keto-3-deoxyoctonate ammonium salt
Man	Mannose
MP	Mannoproteins
PRAG	Polysaccharides Rich in Arabinose and Galactose
PST	Total Polysaccharides Families
RG-II	Rhamnogalacturonan type II
Rha	Rhamnose
Xyl	Xylose
