# Supplementary material for: Effect of Sonication Treatment and Maceration Time in the Extraction of Polysaccharide Compounds during Red Wine Vinification
Source: Molecules. 2021 Jul 23;26(15):4452. doi: 10.3390/molecules26154452 (PMC8348054; doi:10.3390/molecules26154452)
Supplement: Supplementary file 1 [file molecules-26-04452-s001.zip › molecules-1276781-supplementary.pdf]

Table S1. Standard enological parameters of must, must-wines at the end of maceration and wine samples[a]

Parameter[b]	Must[c]		Must-wine[c]					Wine[c]				
	C-M	S28-M	CMF-2d	S28MF-2d	CMF-3d	S28MF-3d	CMF-7d	CW-2d	S28W-2d	CW-3d	S28W-3d	CW-7d
Densidity	1.120 a	1.120 a	1.110 c	1.108 c	1.084 b	1.079 b	1.008 a	0.992 a	0.993 a	0.992 a	0.992 a	0.993 a
Alcohol								15.72 b	15.67 ab	15.52 ab	15.70 ab	14.92 a
TPI	18.29 a	32.07 b	35.25 a	46.06 b	44.73 b	68.25 c	67.85 c	32.93 a	42.60 b	44.01 b	54.63 c	63.83 d

[a]Average of the three measurements. Different letters in the same line indicates statistically differences (p < 0.05). Lower-case letters compare separately musts, must-wines and wines.

[b] Densidity: g/L; Alcohol: % ethanol by volume at 20°C; TA: titratable acidity as g of tartaric acid equivalents/L; VA: volatile acidity as g acetic acid/L; TPI: total phenol index; CI.: color intensity as sum of absorbances at 420, 520 and 620 nm.

[c]C-M, control must; S28-M, must with sonicated grapes at 28 kHz; CMF-2d, control must-wine with two days maceration; S28MF-2d, 28 kHz-treated must-wine with two days maceration; CMF-3d, control must-wine with three days maceration; S28MF-3d, 28 kHz-treated must-wine with three days maceration; CMF-7d, control must-wine with seven days maceration; CW-2d, control wine with two days maceration; S28W-2d, 28 kHz-treated wine with two days maceration; CW-3d, control wine with three days maceration; S28W-3d, 28 kHz-treated wine with three days maceration; CW-7d, control wine with seven days maceration.
